# Commercial app use linked with sustained physical activity in two Canadian provinces: a 12-month quasi-experimental study

**DOI:** 10.1186/s12966-020-00926-7

**Published:** 2020-02-25

**Authors:** Marc Mitchell, Erica Lau, Lauren White, Guy Faulkner

**Affiliations:** 1 Faculty of Health Sciences, School of Kinesiology, Arts & Humanities Building, Western University, Room 3R12B, London, Ontario N6A 5B9 Canada; 2 Carrot Insights Inc., Toronto, ON Canada; 3 University of British Columbia, Vancouver, BC Canada

**Keywords:** Public health, Behavioural economics, Mhealth, Rewards, Physical activity

## Abstract

**Background:**

Top tier commercial physical activity apps rarely undergo peer-reviewed evaluation. Even fewer are assessed beyond six months, the theoretical threshold for behaviour maintenance. The purpose of this study was to examine whether a multi-component commercial app rewarding users with digital incentives for walking was associated with an increase in physical activity over one year.

**Methods:**

This 12-month quasi-experimental study was conducted in two Canadian provinces (*n* = 39,113 participants). Following a two-week baseline period, participants earned digital incentives ($0.04 CAD/day) every day they reached a personalized daily step goal. Mixed-effects models estimated changes in weekly mean daily step count between the baseline period and the last two recorded weeks. Models were fit for several engagement groups and separately by baseline physical activity status within engagement groups.

**Results:**

Nearly half of participants (43%) were categorized as physically inactive at baseline (fewer than 5000 daily steps), and 60% engaged with the app for at least six months [‘Regular’ (24–51 weeks of step data) or ‘Committed’ sub-groups (52 weeks)]. Weekly mean daily step count increased for physically inactive users regardless of engagement status (*P* < .0001). The increase was largest for ‘Regular’ and ‘Committed’ participants—1215 and 1821 steps/day, respectively. For physically active participants, step count increases were only observed in the ‘Committed’ sub-group (*P* < .0001). Effect sizes were modest-to-medium depending on the sub-group analyzed.

**Conclusions:**

A commercial app providing small but immediate digital incentives for individualized goals was associated with an increased weekly mean daily step count on a population-scale over one year. This effect was more evident for physically inactive and more engaged participants.

## Introduction

Despite the health benefits of habitual moderate-vigorous physical activity (PA), [1–3] global rates are precipitously low [4, 5]. For good reason too—exercise is hard and our built environments discourage it [6]. New research, though, suggests health benefits are not just reserved for higher-intensity, harder-to-achieve moderate-vigorous PA, the traditional public health focus [7]. Light*-*intensity PA like walking has beneficial effects as well including lower mortality [8, 9]. From a behavioural perspective, regular participation in less strenuous light-intensity PA may be more attainable on a population level. This perspective was adopted in the latest US Physical Activity Guidelines which stress that some PA is better than none—shifting somewhat from the “at least” 150 moderate-vigorous PA minute/week message [10]. To achieve bold global physical inactivity reduction targets (15% by 2030) the World Health Organization recently singled out digital innovation (e.g., smartphone-based programmes) as an important component of a broad “systems-based” solution in their Global Action Plan on Physical Activity 2018–2030 [11]. To capitalize on the steady growth of the smartphone-based mobile health application (mHealth app) market evaluations of commercial apps that promote any intensity PA are needed [12].

This year more than 2.5 billion people worldwide own a smartphone [13]. The number of mHealth apps published in the major app stores continues to rise with 325,000 published in 2017, up 34% from the previous year [14]. This increase in part reflects evolving smartphone capabilities (e.g., built-in accelerometers, geo-location). Access to built-in accelerometer data in particular [15] has transformed PA promotion. For the first time, the majority of adults (approaching 90%) in the US and Canada, for example, carry a PA monitoring device (i.e. a smartphone accelerometer) most of the time [13]. This presents an unprecedented opportunity to deliver more precise public health interventions and bridge well-worn PA divides (e.g., gender PA gaps) [16] using instantaneous PA data to set *and adjust* realistic PA goals, provide immediate feedback, link users with friends to support long-term change, and so on. Not surprisingly, PA apps make up the bulk of all mHealth apps (30%, or roughly 100,000 apps) [17]. Unfortunately, low PA app engagement leading to small effects and little sustainability have been industry hallmarks [17–19].

A 2016 systematic review [18] and a 2019 meta-analysis [20] of studies using apps to improve PA found that few stand-alone app interventions reported positive effects. Another recent meta-analysis [21] and systematic review [19] on the other hand found that app-based interventions increased PA. The still limited number of RCTs in this area (*n* < 10), due in part to the rapid pace of app development and rollout, may help explain the discrepancies [19, 20]. To enhance our understanding of this rapidly evolving field non-RCT alternatives (e.g., quasi-experimental designs) are needed [19, 22, 23]. Longitudinal designs in particular are warranted given the majority of studies do not exceed three months [19–21] even though sustained PA is needed to attain many of the purported health benefits [1]. Rigorous quasi-experimental evaluations of top tier commercial apps (i.e. the top 2% of all apps reporting more than 500,000 Monthly Active Users, MAUs—at least one app view per month) [14] may provide especially valuable insight in a promising field where attrition is unfortunately the norm. Among the 15 studies included in the Petersen et al. (2019) review of PA apps only five examined commercially available ones (e.g., Fitbit, ‘Zombie, Run!’). Other studies have examined the Pokémon Go! [24] and Sweatcoin [25] apps, though important limitations preclude strong conclusions (e.g., unrepresentative samples).

**Fig. 1:**
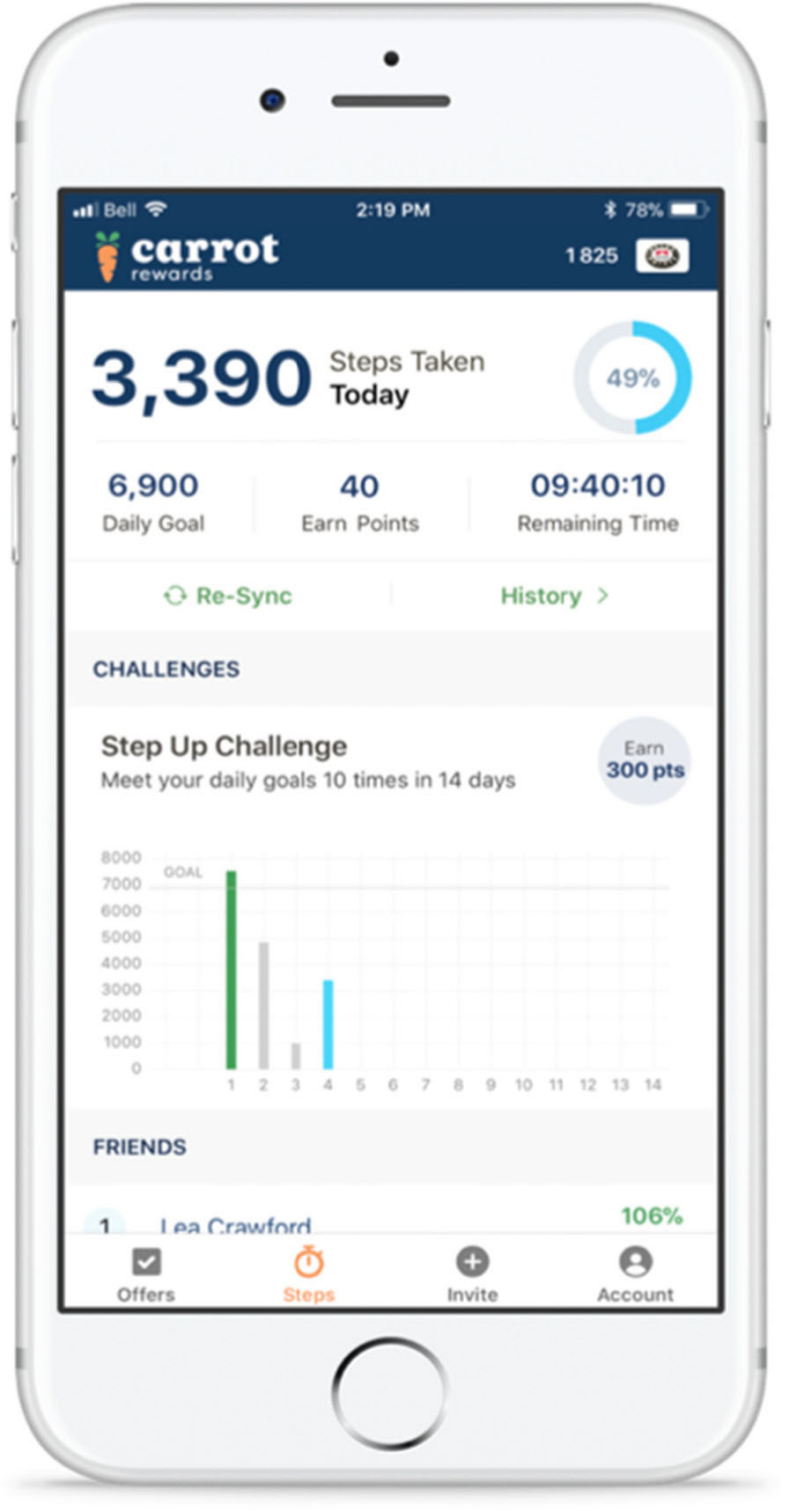
Carrot Rewards app “Steps” walking program screenshot

The Carrot Rewards app, created by a private company with support from the Public Health Agency of Canada, [26] presents a unique opportunity to explore the long-term effectiveness of a top tier commercial app. Carrot Rewards was a popular Canadian app (i.e. 1.3+ million downloads, 500,000+ MAUs as of May 2019) leveraging gamification elements [27] and concepts from behavioural economics [28] and self-determination theory [29] to reward users with digital incentives (i.e. loyalty points redeemable for consumer goods like movies or groceries) for engaging in healthy behaviours such as walking. The multi-component Carrot Rewards “Steps” walking program (which included goal setting, biofeedback, daily/weekly incentives, etc.; Fig. 1) provided very small incentives for individualized daily step goal achievements. A three-month “Steps” evaluation was published in 2018 [30]. In this quasi-experimental study of users living in two Canadian provinces, Mitchell et al. (2018) found average daily step count increased by 5% between baseline and the three-month assessment (115.70 steps; *P* < .001). A more pronounced 32% increase was observed among highly engaged, physically inactive users (1224.66 steps; *P* < .001). As is commonly reported [18, 19] behavioural decay was noted in the later part of the three-month evaluation.

Since the effectiveness of PA apps have been shown to wane over time, the primary objective of this study is to examine the impact of the Carrot Rewards app over a longer 12-month period. Longitudinal designs such as this are especially important in a Canadian context, and for other countries, where inclement weather can dampen PA behaviours. Determining whether PA changes are moderated by app engagement is an important secondary objective.

## Methods

### Study design, setting and participants

A 12-month quasi-experimental (pre/post single group) study design was used. The free Carrot Rewards app was made available to British Columbia (BC) and Newfoundland and Labrador (NL) residents on the Apple iTunes and Google Play app stores on March 3 and June 13, 2016, respectively. Only users enabling the “Steps” walking program (i.e. allowing the app to access their step data) during the June 13 to July 10, 2016 recruitment period were included in the study. Additional background information, including recruitment details and theoretical underpinnings are published elsewhere [30]. Carrot Rewards was discontinued in June 2019 due to a lack of funding [31]. The *Strengthening the reporting of observational studies in epidemiology* (STROBE) *statement* checklist for cohort studies is provided (Additional file 1). The University of British Columbia Behavioural Research Ethics Board approved this study (H17–02814).

### Intervention: “Steps” walking program

Once enrolled in the walking program users were instructed to “wear” their smartphone or Fitbit™ as much as possible during the two-week baseline period. After the baseline period, users could begin to earn incentives for reaching individualized daily step goals (Fig. 1). To set users’ first personalized daily step goal, 1000 steps was added to their baseline daily step count average. The incentives for daily achievements were worth $0.04 CAD in loyalty points. After roughly four weeks of earning daily rewards users could then begin to earn a $0.40 CAD bonus for reaching their daily goal 10 or more times in a 14-day period, called a “Step Up Challenge” (Fig. 1). For users who successfully completed a “Step Up Challenge” a new higher daily step goal was provided. For unsuccessful users, the previous goal persisted. Over the course of the 12-month evaluation, participants could earn a *maximum* of $25.00 CAD in points. Like with many apps the intervention evolved over time (Table 1). For more app design detail, completed Mobile App Rating Scale (MARS self-score 4.23/5; for understanding app quality, aesthetics and functional appeal) [32] and App Behavior Change Scale (ABACUS self-score 4.5/5; for measuring potential to change behaviour) [12] are provided (Additional files 2 and 3).

**Table 1:** Carrot Rewards “Steps” walking program timeline and evolution

Date	Milestone	Description
June 13, 2016	“Steps” Program Launches	“Steps” launches in British Columbia and Newfoundland & Labrador
July 8, 2016	First Goal Modified	1000 step increase initially added to baseline mean to set daily goal is removed
July 28, 2016	“Step Up Challenge” Launches	Users earn bonus reward for achieving 10 or more daily goals in 14 days
September 20, 2016	FitBit Integration	Users given option of tracking steps with any FitBit device
October, 2016	Mid-Week Bonuses	For the month of October users earn 2, 5, or 10 times the points for reaching daily goals on Wednesdays
February 9, 2017	‘Smart Goals’	Adaptive goal setting introduced where users’ goals re-calculated every 2–4 weeks using data from last 30 days
July 9, 2017	Evaluation Period Ends	12-month evaluation period concludes

### Outcome measure

The primary outcome was mean daily step count as measured by either built-in smartphone accelerometers or Fitbit trackers (i.e. iPhone 5S or higher [26.21% of users], Android devices [42.78%]), Fitbit^TM^ devices [4.43%], Unknown device [26.59%]). In recent validation studies, the iPhone step counting feature (version 6 or newer), as well as those for Android smartphones (e.g., HTC) and Fitbit trackers (e.g., wrist-worn Flex) were accurate in laboratory and field conditions [33–35]. While wrist-worn activity trackers may record more daily steps than smartphone-based accelerometers (e.g., given wear time differences), given the small proportion of participants using Fitbit we decided not to examine effects by device.

### Co-variates

The majority of demographic variables used to describe the study sample were self-reported (e.g., age, gender). Median personal income was inferred by linking user postal codes with census data (i.e. 2011 National Household Survey) at the Local Health Area level (89 LHAs) in British Columbia and Regional Health Authority level (four Regional Health Authorities) in Newfoundland & Labrador. Baseline daily step count set date was included as a co-variate in our analyses as well to adjust for potential seasonal effects.

### Analyses

Statistical analysis was performed using R 3.3.0 .68 Mavericks build (7202). Two sets of analyses were used to estimate changes in mean daily step count over the intervention period. In our primary analysis, and similar to our first 12-week examination of “Steps”, we estimated changes in mean daily step count between baseline and the last two recorded weeks. We included participants who had valid baseline data (four or more days with step counts in acceptable range, between 1000 and 40,000, during the 14-day baseline period) and at least one other valid week (at least four valid days in a week) between study week 1 and 52 (88% of those enrolling in “Steps” during recruitment period; 39,113/44373) in the analysis. We did not remove any cases or perform imputation to account for missing data since these approaches did not influence results in our 12-week analyses [30]. Time was coded as a three-level categorical variable (baseline = 0, second last recorded week = 1, and last recorded week = 2). Mixed-effects models were used to examine whether there were significant changes in mean daily step counts between baseline and the last two recorded weeks. A full model was fit that included time (with baseline as one of the three levels), demographics, and baseline set date as fixed effects along with participant as a random effect. A post hoc contrast was then estimated for the difference between the average of the last two recorded weekly average daily step count and baseline. A reduced model was also fit that included time and the baseline set date as the fixed effects.

In our secondary analysis, we estimated the longitudinal change in weekly record of mean daily step count across all 52 weeks. The purpose of this analysis was to illustrate how changes in weekly average daily step count varied across one year. The outcome variable was weekly mean recorded daily step count. Time was coded as a categorical variable (baseline = 0, week 1 = 1, …, week 52 = 52) to allow for the non-linear trajectory of daily step count. A mixed-effects model was used to examine on average the overall magnitudes of change across weeks. We fit a full model that included time with demographic variables, baseline set date, and baseline daily step count as fixed-effect co-variates and participant as a random effect.

As results from our 12-week analyses indicated that engagement and PA status had significant moderating effects on changes in weekly mean daily step counts over time, we fit all models separately for several engagement groups and then separately for physically active and physically inactive participants within each engagement group. Four engagement groups were formed based on number of weeks with four or more days of valid step count data: ‘Limited’ users: 1–11 weeks, ‘Occasional’ users: 12–23 weeks, ‘Regular’ users: 24–51 weeks and ‘Committed’ users: 52 weeks. An app view would trigger daily step count data retrieval for the previous four weeks. Two PA status categories were formed as defined by Tudor-Locke et al. [36]: physically inactive users = baseline mean steps per day less than 5000; physically active users = baseline mean steps per day 5000 or more. Cohen’s f2 for local effect sizes of weekly mean daily step counts within the mixed-effects models were calculated, with f^2^ ≥ 0.02, f^2^ ≥ 0.15, and f^2^ ≥ 0.35 representing small, medium, and large effect sizes, respectively [48]. Statistical significance of fixed effects was assessed using Wald statistics. Statistical significance levels were set at *P* < 0.05.

## Results

### Baseline characteristics

Of the 39,113 participants, the mean age was 33.7 ± 11.6 years and 66.0% (25,809/39,113) were female (Table 2). Other incentive-based eHealth interventions have reported similar demographic profiles, with females in particular being more likely to adopt with digital wellness interventions in general [26, 27]. Sixty percent of users (23,505/39,113) engaged with the app for at least six months being categorized as either ‘Regular’ or ‘Committed’. The mean personal median income was $29,517 CAD, lower than the 2014 BC and NL means of $31,610 and $30,450 CAD, respectively [38]. Mean daily step count at baseline was 5560 steps per day. Nearly half of users (42.46%, 16,606/39,113) were categorized as physically inactive. Average incentive amount earned over the course of the year ranged from $5.88–$10.83 CAD depending on participant’s age, gender and province.

**Table 2:** Baseline characteristics of study participants by engagement group, and Canadians in general

	Limited (< 12 weeks)	Occasional (12–23 weeks)	Regular (24–51 weeks)	Committed (52 weeks)	Total	Canadian Population
Sample size	8349	7259	15,557	7948	39,113	35,151,728
Age, years (mean, SD)	33.2 (11.9)	33.0 (11.4)	33.7 (11.4)	35.0 (11.6)	33.7 (11.6)	40.6 (median)
Gender, (% female)	65.8	66.8	67.2	63.1	66.0	50.4
Province, (% BC)	69.3	69.7	74.2	76.8	72.8	13.2
Median Personal Income, (mean, SD; thousand CAD/year)	29,560 (3880)	29,519 (3936)	29,452 (4019)	29,597 (4130)	29,517 (3998)	35,680
Steps per day, baseline mean, (SD)	5349 (2657)	5443 (2693)	5433 (2642)	6136 (2907)	5560 (2726)	8965^a^

Note. *SD* standard deviation, *CAD* Canadian ^a^ Colley et al. 2011 [37]

*BC* British Columbia

### Difference between the last two recorded weeks and baseline

As presented in Table 3, the intervention effect was more pronounced among physically inactive and more engaged sub-groups. For the engagement sub-group analysis, average daily step counts from baseline to last recorded weeks significantly increased in ‘Regular’ and ‘Committed’ users, but significantly decreased in ‘Limited’ and ‘Occasional’ users. Cohen’s f^2^ statistic indicated that the effect was small (0.0563) for ‘Committed’ users and modest for the other engagement groups. PA status also had a significant moderating effect. We observed a significant increase in daily step counts from baseline to the last two recorded weeks in physically inactive participants across engagement groups. However, significant and small decreases were noted for all physically active participants except those categorized as ‘Committed’ (Table 3).

**Table 3:** Changes in mean daily step counts between baseline and the last two recorded weeks stratified by engagement group and physical activity status within engagement group, least-square means (LSM) and 95% confidence intervals

	Differences^a^ (Average of last two recorded weeks - Baseline)	Cohen’s f^2^
*Engagement groups*		
Limited users	− 392.3 (− 439.9 to − 344.7)	0.0173
Occasional users	− 473.2 (− 527.4 to − 418.9)	0.0211
Regular users	448.8 (407.9 to 489.7)	0.0156
Committed users	884.6 (824.8 to 944.4)	0.0563
*Physical activity status within engagement group*		
Physical inactive, Limited users	388.6 (333.6 to 443.6)	0.0287
Physically inactive, Occasional users	435.5 (372.2 to 498.9)	0.0306
Physically inactive, Regular users	1215 (1163 to 1266)	0.1617
Physically inactive, Committed users	1821 (1739 to 1902)	0.3140
Physically active, Limited users	− 957.9 (− 1027 to − 888.3)	0.0818
Physically active, Occasional users	− 1141 (− 1219 to − 1062)	0.1011
Physically active, Regular users	−161.1 (− 220.4 to −101.7)	0.0017
Physically active, Committed users	262.3 (181.4 to 343.3)	0.0059

*Note: LSM have been averaged over the levels of gender and province and assumed an average level for all of the other continuous demographic variables included in the model. Cohen’s f*^*2*^ *≥ 0.02, f*^*2*^ *≥ 0.15, and f*^*2*^ *≥ 0.35 represents small, medium, and large effect sizes, respectively*

^a^The difference between baseline and the average of last two recorded weeks were statistically significant at *P* < .0001 for all sub-group analyses

### Longitudinal changes in weekly mean daily step counts

**Fig. 2:**
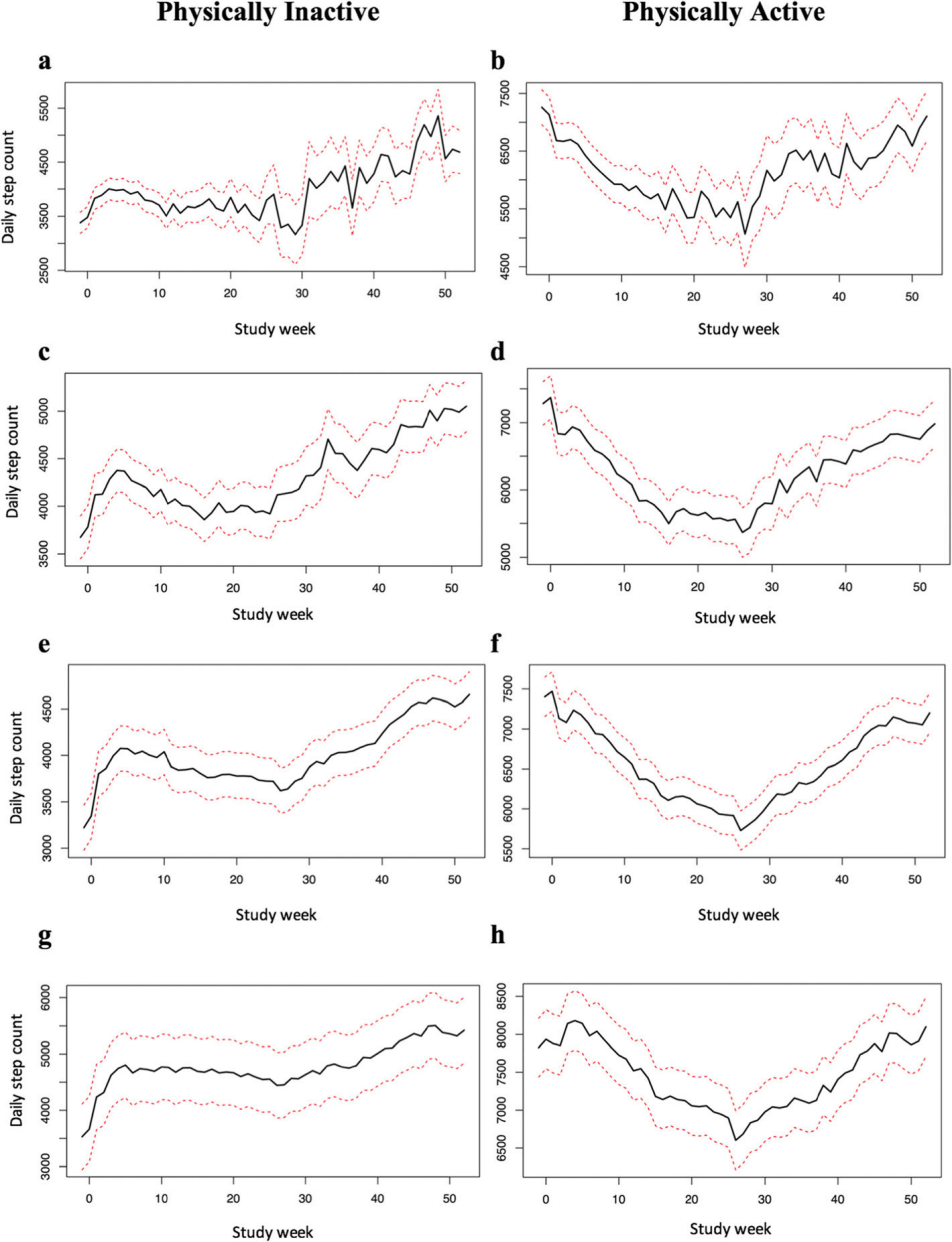
Longitudinal changes in weekly recorded mean step counts by physical activity status and engagement group, with 95% confidence intervals (dotted line). Models adjusted for baseline set date and baseline daily step counts. **a-b**, ‘Limited’ users; **c-d**, ‘Occasional’ users; **e-f,** ‘Regular’ users; **g-h**, ‘Committed’ users

For all models, we observed significant effects of time and baseline daily step count. The overall pattern of change in weekly recorded steps differed by PA status regardless of engagement group. In Fig. 2, we illustrate the longitudinal change in weekly recorded mean steps by engagement group within PA status. For physically inactive users, weekly recorded mean step counts were above baseline levels in most weeks. The magnitude of increase varied across weeks, reaching a peak at week 4 (July/August, summer season), followed by a modest PA decline and plateau from week 5 to week 27 (December, winter season), with a generally positive trend thereafter. For physically active users, weekly recorded mean step counts were below baseline in most weeks. A steeper decline from week 5 to 27 was noted for these physically active users, with daily step count increases in the remaining weeks. The dip in average daily step count in the weeks leading up to the shortest day of the year (Winter Solstice on December 19, 2016; weeks 23 to 26) was generally more pronounced in physically active compared to physically inactive users.

## Discussion

### Main finding

In this large quasi-experimental study examining the impact of the Carrot Rewards app on objectively-assessed PA over one year, we observed a significant intervention effect in the physically inactive users regardless of engagement status. The increase was largest for ‘Regular’ and ‘Committed’ users—1215 and 1821 steps per day, respectively. The clinical implications of these increases are important especially when one considers that the majority of the health benefits of PA (e.g., systolic blood pressure, glycemic control improvements) are reserved for inactive adults who become a little more active [9, 39]. From a public health perspective, a 1% reduction in the number of Canadians classified as physically inactive would yield annual healthcare savings of $2.1 billion CAD [40]. If we generalize our findings to the larger Carrot Rewards user base (1,046,185 users as of April 2019) then we estimate that the number of Canadians classified as physically inactive was reduced by 0.3% (about 100,000 Canadians).

### Secondary findings

A dose-response relationship was evident with more favourable effects observed for more engaged users, irrespective of PA status. This highlights the importance of maximizing engagement with evidence-based mHealth intervention designs such as the ones included in the recent and very useful App Behaviour Change Scale, or ABACUS, checklist (e.g., Does the app allow for the setting of goals? Does the app have prompts for activity? Does the app provide material or social incentive?) [12]. Carrot Rewards’ high ABACUS rating (self-score = 4.5/5; see Additional file 3) may partly explain why 60% of the study sample used the app for at least six months (that is, those classified as ‘Regular’ or ‘Committed’)—the theoretical threshold of behaviour maintenance [41]. Maintaining fidelity to two behaviour change theories in particular also likely fostered high initial and sustained engagement (e.g., behavioural economics, by offering rewards instantaneously; self-determination theory, by providing realistic and personalized goals). Few studies in this field have reported engagement metrics, and even fewer have examined the interaction between engagement and health behaviours/outcomes [18–20]. Those that have suggest that intervention exposure is imperative and that greater engagement usually yields larger effects [20].

In addition, our longitudinal analysis illustrates great variation in PA over the course of a year. This is consistent with previous research that found seasonality impacts PA patterns in Canada [42]. Notably, seasonality impacts on PA vary across Canadian provinces with season being a stronger predictor of PA in BC then it is in NL. PA fluctuations over the year should therefore be considered in the refinement of PA apps in the future (e.g., PA goals could be reset in the winter to attenuate declines in steps rather than increasing steps). In addition, the longitudinal analysis partly confirms intervention effects among physically inactive users. Weekly mean daily step counts increased above baseline in most weeks in inactive users, but decreased below baseline in most weeks for active participants. In particular, the winter-time drop was less remarkable in ‘Committed’ physically inactive compared to physically active users as found in a recent Pokémon Go! app analysis where ‘players’ (vs. ‘non-players’) did not experience winter-time step count reductions [43]. This suggests that the intervention *may* have protected against winter-related PA decreases. Future study with a comparison condition is needed for verification.

### Similar studies

Our findings are comparable to those of a recent meta-analysis of RCTs testing PA incentives delivered using smartphone/wearable technology (*n* = 12). In this study Mitchell et al. (2019) concluded that incentives increased mean daily step counts for short and long duration interventions by 607 steps [44]. Sub-group meta-analyses suggested physically inactive adults are especially sensitive to incentive intervention and that PA increases do not necessarily wane for longer interventions, consistent with what was found here. There was little to suggest physically active participants, other than ‘Committed’ ones, increased their steps over the year. In addition, our results build-on the efficiencies noted in the meta-analysis. That is, reward sizes needed to stimulate PA have dropped considerably in recent years due in part to technological advances that make it easier to track and reward activity, and stronger application of behavioural economics concepts. By offering digital incentives instantaneously Carrot Rewards drove incentive cost down (to pennies a day) by exploiting two behavioural economics concepts in particular: (a) the human tendency to prefer payoffs close to the present time (“present bias”) and (b) the tendency for people to equate larger numbers (i.e. the points used in this case) with greater value (“numerosity”).

On the other hand, few rigorous evaluations of top tier PA apps have been published [19]. Sweatcoin, a popular UK-based app (30+ million downloads globally) that converts step counts into a virtual currency, is a notable and relevant exception [25]. In a nine-month observational study (*n* = 5892), Elliot et al. (2019) determined in the six months following registration that daily step count increased by 18.7% (roughly 1200 steps) compared to baseline. While this study had several strengths (e.g., assessed long-term impact of a commercial app on objectively-measured PA) the main findings must be interpreted with caution. In particular, the Elliot et al. (2019) analysis included only very engaged users (opened app in last seven days) with complete data sets—unlike this study in which *all* users signing-up for the “Steps” program during the evaluation period and with one other valid week were included. It is unclear whether analyses of this highly engaged sub-sample—only 5892 users out of more than 30+ million were included—can be generalized to the broader user base. In addition, with the majority of the sub-sample in the Sweatcoin study registering in the winter it is unclear if effects are due to typical seasonal PA fluctuations. As well, smartphone wear time during the pre-registration period was not optimized unlike with the present study where users were encouraged to “wear” their smartphones as much as possible during the baseline period. In terms of effect magnitudes, the results of the present study generally align with those of Elliot et al. (2019) with roughly 500 to 1500 daily step count increases observed depending on the sub-group analysed. Notably, and consistent with our findings, physically inactive Sweatcoin users responded the most.

### Limitations

Our results should be interpreted with caution in light of some limitations. First, the internal validity (i.e. the extent to which PA increases were *caused* by Carrot Rewards) of our findings are limited by the absence of an equivalent control group. To address this limitation, we defined a pre-intervention time period (the two-week baseline period), distinct from the intervention, to reflect the counter-factual in this quasi-experimental setting [45]. The anticipated daily step count increase from the pre-intervention baseline period to intervention Weeks 1 and 2 was observed (Fig. 2) suggesting “Steps” boosted PA when introduced. Analysis-phase strategies were also employed to improve internal validity [45]. All models adjusted for key demographics variables, baseline set date and baseline step count, and accounted for measurements nesting within individuals. As well, a clear dose-response relationship between engagement and PA provides further support for the main conclusion that Carrot Rewards, when used above a threshold level, is associated with an increase in PA. A rival hypothesis may be that participants simply started carrying their smartphones more. The challenge of disentangling “wear time” from actual daily step count increases is a limitation of this and other similar studies [46]. A second limitation is that complete data sets (data for all 52 weeks) were only available for 20% of study participants. Unlike for the ‘Committed’ users (for whom we know the last two recorded weeks occurred exactly one year after baseline because data for all 52 weeks were available) it is not exactly clear when the last two recorded weeks for the other engagement groups occurred given their incomplete data sets. Data could have been recorded during a calendar month/season that was different from baseline, for instance. Third, it is not known at what intensity any extra steps were accumulated. Collecting step count data on a minute-by-minute basis in the future may help establish step cadences that could be classified as at least moderate intensity. Similarly, measuring key clinical variables (e.g., A1C) in at least a sub-sample of users may help establish the expected clinical benefits of app use and facilitate the ‘prescription’ of such an app and inform important health economic analyses.

## Conclusion

A multi-component commercial app providing very small (i.e. $5-$10 CAD per person per year) but immediate digital incentives for individualized goals was associated with an increase in weekly mean daily step count on a population-scale over one year. This was particularly the case for physically inactive and more engaged users. The clear dose-response relationship between engagement and changes in daily step count reinforces the fundamental importance of engagement in digital health interventions. The high proportion of ‘Regular’ and ‘Committed’ users over one year suggests some success of the Carrot Rewards app in that regard.

## Supplementary information

**Additional file 1:** STROBE Checklist.

**Additional file 2:** Mobile Application Rating Scale (MARS) and self-score.

**Additional file 3:** App Behavior Change Scale (ABACUS) and self-score.

## Data Availability

The datasets used and/or analysed during the current study are available from the corresponding author on reasonable request.
