# Supplementary material for: Commercial app use linked with sustained physical activity in two Canadian provinces: a 12-month quasi-experimental study
Source: Int J Behav Nutr Phys Act. 2020 Feb 25;17:24. doi: 10.1186/s12966-020-00926-7 (PMC7043029; doi:10.1186/s12966-020-00926-7)
Supplement: Supplementary file 3 — Additional file 3: App Behavior Change Scale (ABACUS) and self-score. [file 12966_2020_926_MOESM3_ESM.pdf]

Original Paper

The App Behavior Change Scale: Creation of a Scale to Assess the Potential of Apps to Promote Behavior Change

Fiona H McKay, BSc, MPH, PhD; Sarah Slykerman, BHS, MHP, GradDipEd; Matthew Dunn, BA, PhD

School of Health and Social Development, Deakin University, Burwood, Australia

Corresponding Author:
Fiona H McKay, BSc, MPH, PhD
School of Health and Social Development
Deakin University
Burwood Hwy
Burwood, 3125
Australia
Phone: 61 392517183
Email: fiona.mckay@deakin.edu.au

Abstract

Background: Using mobile phone apps to promote behavior change is becoming increasingly common. However, there is no clear way to rate apps against their behavior change potential.

Objective: This study aimed to develop a reliable, theory-based scale that can be used to assess the behavior change potential of smartphone apps.

Methods: A systematic review of all studies purporting to investigate app's behavior change potential was conducted. All scales and measures from the identified studies were collected to create an item pool. From this item pool, 3 health promotion exerts created the App Behavior Change Scale (ABACUS). To test the scale, 70 physical activity apps were rated to provide information on reliability.

Results: The systematic review returned 593 papers, the abstracts and titles of all were reviewed, with the full text of 77 papers reviewed; 50 papers met the inclusion criteria. From these 50 papers, 1333 questions were identified. Removing duplicates and unnecessary questions left 130 individual questions, which were then refined into the 21-item scale. The ABACUS demonstrates high percentage agreement among reviewers (over 80%), with 3 questions scoring a Krippendorff alpha that would indicate agreement and a further 7 came close with alphas >.5. The scale overall reported high interrater reliability (2-way mixed interclass coefficient=.92, 95% CI 0.81-0.97) and high internal consistency (Cronbach alpha=.93).

Conclusions: The ABACUS is a reliable tool that can be used to determine the behavior change potential of apps. This instrument fills a gap by allowing the evaluation of a large number of apps to be standardized across a range of health categories.

(JMIR Mhealth Uhealth 2019;7(1):e11130) doi:10.2196/11130

KEYWORDS

apps; smartphone; mobile phone; mobile app; scale development; rating

Introduction

The delivery of psychological and public health interventions through technology is becoming an increasingly common way to prevent illness and promote health. Smartphones and tablets are well positioned to play a role in such interventions as they offer functionalities and opportunities for personalization through the widespread availability of a range of mobile phone apps [1]. Apps play an important role in the management of illness and are a low-cost, easy avenue for the promotion of health and well-being [2-4]. In 2017, there were 325,000 *health* apps across the 2 most common app platforms: Google Play and iTunes [5]. This includes apps that have been developed to assist patients in the management of a range of diseases and conditions, including diabetes mellitus type 1 or 2 [1,6], pain management [7,8], the promotion of increased physical activity [9,10], improve nutrition [11,12], and the promotion of improved mental health [13,14].

Although research investigating mobile phone–based technology over recent years has shown that short message service (SMS) text message–based interventions can have a positive impact on sexual health knowledge [15] and that most health interventions can benefit from some form of phone-based

XSL•FO
RenderX

activity [16], research into the effectiveness of health behavior change through apps is in its infancy, and there is no clear consensus in the research around which specific features of apps can assist in behavior change. Content analyses of apps have identified some features that may promote health behavior change in apps for smoking cessation [17], alcohol reduction [18,19], and physical activity [20,21]. However, most apps only contain a few features that could be considered to have the potential to change behavior [22]. Features that have been found to promote health behavior change include the ability to provide direct advice about behavior change and track behaviors [17] or provide information on the consequences of continuing with the behavior [19]. Conversely, those studies that have found apps to be lacking in health behavior change features have highlighted the absence of individual tailoring such as personalized notifications or the collection of background information, for example, using global position system data to identify when a person might be at a high-risk area for alcohol use [18] or simply asking a user to set a smoking quit date [17].

Studies that report on user outcomes or experiences of apps have had similarly mixed results. One systematic review that investigated the role of apps and other digital media in physical activity and diet as it relates to cancer survivorship found an overall increase in minutes of physical activity with use of the app, but mixed evidence for improved diet, and no improvement for secondary outcomes such as a reduction in anxiety or depression [23]. A recent study investigating the role of apps in improving mental health found that after 30 days of app use, mental well-being improved in those using 1 of 3 mental well-being apps tested and those using 1 of the 3 apps tested showed improvements in depression. None resulted in improvements in anxiety [24]. A systematic review and meta-analysis of studies that employed a smartphone app to increase physical activity found that the use of apps could result in significant changes to body weight and body mass index; however, nonsignificant results were identified in changes to physical activity [25].

Alongside this growing body of interest in the identification of apps that may play a role in behavior change [26,27] is an increasing body of research that seeks to first understand the features of apps that may play a role in behavior change and then to measure and classify these features [28-30]. Common among these studies is an aim to identify features that employ best practice to allow health practitioners to better inform consumers and patients of the apps most suited to their needs. The ability of practitioners to give this advice is predicated on the ability of researchers to effectively classify and evaluate apps suitable for the most common health conditions through a reliable and valid measurement tool.

As described by McKay et al [31], many studies investigating the potential of apps to change behaviors have employed a behavior change taxonomy (either the CALO-RE or 26 or 93 item taxonomy) for the rating and categorization of apps [10,20-22]. The aim of the systematic review undertaken by McKay et al [31] was to investigate ways in which researchers evaluate the potential health behavior change of apps to identify any current best practice approaches. Instruments identified in the review were created to investigate the behavior change potential of Web- and text-based health interventions [32]. The techniques present in these instruments have been identified in a range of studies and then linked back to behavior change potential. Most notable are by Abraham and Michie [33], who suggested a number of behavior change techniques common to many health behavior theories. Michie et al [34] identified 5 techniques present in physical activity and dietary interventions: self-monitoring, intention formation, specific goal setting, review of behavioral goals, and feedback on performance, finding that interventions that included self-monitoring with at least one other technique were responsible for the largest effect size [34]. These findings are supported by other work suggesting that self-monitoring is useful for increasing physical activity and improving diet for those who were overweight with comorbidities [35], with other work suggesting that self-monitoring is one of the strongest predictors of weight loss [36] and can also assist in decreasing alcohol consumption [37].

App-based studies that have employed these taxonomies have found apps to be lacking in the identified characteristics of a good behavior change intervention. For example, in an investigation of 166 apps that encourage medical adherence against 93 behavior change techniques, Morrissey et al [22] found most apps contained between 0 and 7 techniques, with the most common technique identified being *action planning*, where users are able to set a reminder to take medication at a specific time every day, and set prompts or cues, typically through the setting of an alarm. A total of 2 studies investigated physical activity but found few techniques for behavior change. Direito et al [21] found that most apps contained 8 techniques, most frequently providing instruction, setting graded tasks, and employing self-monitoring, whereas Conroy et al [10] identified 4 or fewer techniques in the physical activity apps they reviewed.

As more practitioners begin to recommend apps to patients for a range of health care needs [38,39], it becomes essential that we have a valid and reliable way to evaluate these apps. Although both valid and reliable, the taxonomies of behavior change theory [33] were designed to evaluate the features of text and Web-based interventions [32,40,41], not for the review of apps. For instance, these taxonomies often feature a large number of items that are closely related, and are theoretically important in behavior change theory, but will often only appear once in an app. For example, the behavior change taxonomy used by Morrissey et al [22] includes 93 items, with each item allocated a score of 1 if present and 0 if absent. Many of these items are similar, for example, there are 11 items categorized as *reward* (including material incentives, material rewards, and nonspecific rewards), all of which are classified separately. For most apps, only one of these items would be present, thus although an app may offer rewards and the benefits that they bring to behavior change, they only offer 1 type means that app would receive a low score in that behavior change category. With increasing knowledge and the growing body of research into app-based interventions, there is a clear need for a purpose-designed app rating system to identify the potential for health behavior change. Although there is 1 scale, the Mobile App Rating Scale (MARS), that is able to describe the functionality of apps, including aesthetics and information

XSL•FO
RenderX

shared [42], there is currently no scale that can measure the potential for behavior change.

Over the past 3 years, the 3 authors of this study have been involved in rating and reviewing apps for the Victorian Health Promotion Foundation (VicHealth) Healthy Living Apps project [43]. The VicHealth Healthy Living Apps project is an annual rating activity using the MARS [42] and CALO-RE [40] scales to provide consumers with a guide to which apps may assist them best in promoting health. This project typically sees up to 400 apps rated annually for their functionality and ability to encourage or promote behavior change in 1 of the following 5 categories: healthy eating, physical activity, tobacco prevention, alcohol harm prevention, and mental well-being. These categories have been chosen as they form the key priority areas of VicHealth and, therefore, are those that are investigated in the VicHealth Healthy Living Apps project [43]. This experience has made clear to the authors that a purpose-designed scale to measure the health behavior change potential is needed for any app review that seeks to recommend apps to the public.

This study aims to develop a reliable, theory-based scale that can be used to assess the behavior change potential of smartphone apps.

Methods

Study Design

The creation of this scale occurred in 4 phases. Phase 1 included a systematic review to identify all scales that have been used to rate the potential of an app to encourage behavior change. Results from this phase were analyzed and developed into a draft tool. Phases 2 to 4 consisted of series of deductive tests. The results of each round of testing were analyzed and incorporated into the next version of the scale until the team could be confident of reliability and validity of the scale. The final version of the scale was shared with a panel of experts for comment and feedback (see Figure 1 for an overview of the study procedure).

Phase 1: Systematic Review to Develop Initial Item Pool

A systematic search of the literature was conducted to gather all published evidence relating to the various ways that apps have been evaluated for behavior change potential to develop an item pool. This search was based on and extended a previous systematic review [31]. A total of 5 databases (Academic Search Complete, CINAHL Complete, E-Journal, MEDLINE Complete, and PsycINFO) were systematically searched. The search was completed on November 17, 2017, with no temporal limitations placed on the search. The search was limited to studies focusing on mobile phones, smartphones, cell phones, and tablets; used apps; and focused on health behaviors previously investigated in the VicHealth Healthy Living Apps project [43]. Search terms were health, wellbeing, preventative health, smok*, nutrition, alcohol, physical activity, or mental wellbeing.

The inclusion criteria comprised studies that evaluated mobile health apps in English, evaluations or reviews of apps targeted at consumers, alone or in addition to health professionals, and studies that evaluated the effectiveness of mobile health apps.

Figure 1. Study procedure. ICC: interclass coefficient; ABACUS: App Behavior Change Scale.

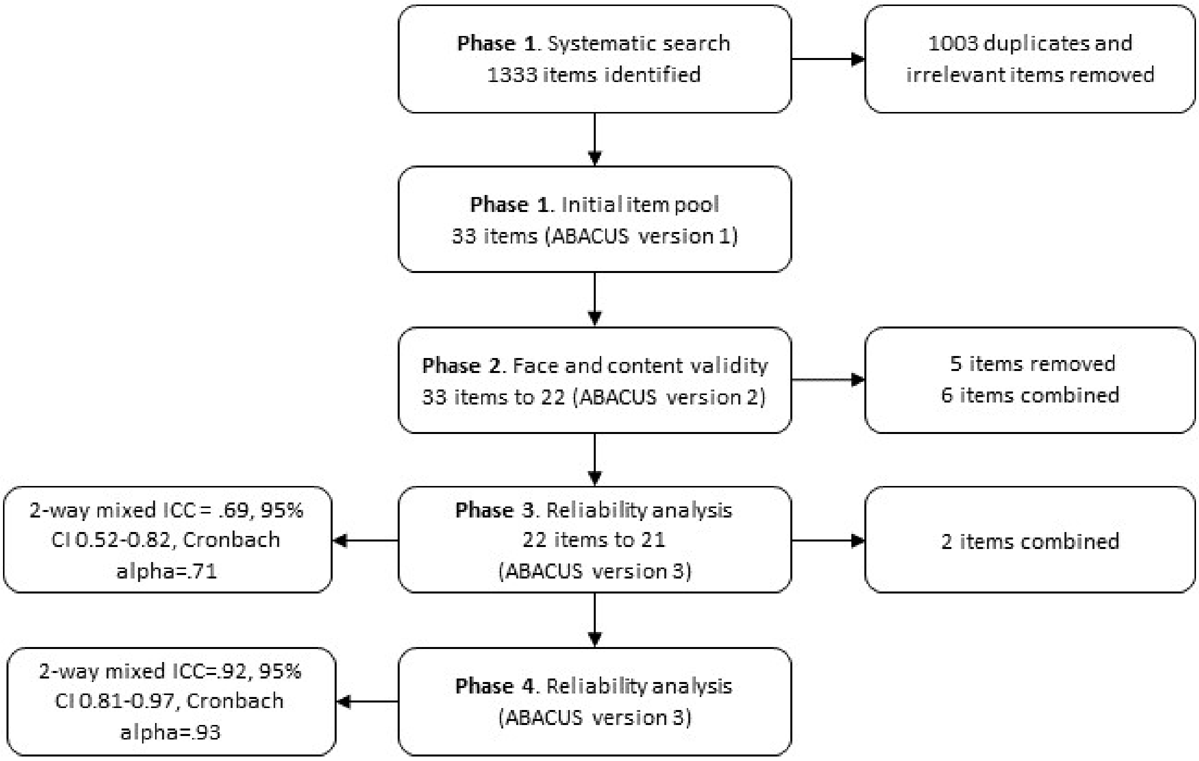

XSL•FO
RenderX

Excluded studies comprised those that evaluated mobile health apps targeted only at health professionals, formative evaluations of mobile health apps, protocols for evaluations, apps that were not publicly or commercially available, studies that reported primarily on the validation of any mobile health app tool (eg, the MARS), and studies of apps not related to health behavior change. The papers were first screened by title and abstract based on the inclusion and exclusion criteria. The full texts of selected papers were then obtained for further assessment for final inclusion.

Phase 2: Face and Content Validity

The initial version of this scale was pilot-tested with 3 physical activity apps. The pilot testing was conducted by 2 experienced reviewers (FM and SS) and allowed the raters to (1) become familiar with the scale and (2) refine the wording of items and create item descriptors and examples.

Following this pilot, the ABACUS version 1 was used to rate the 3 highest rating apps from each of the 5 categories (15 apps in total) from the VicHealth Healthy Living Apps project [43]. To undertake this testing, the reviewers downloaded and became familiar with each app. Similar to other studies [28,42,44], the authors spent approximately 10 to 15 min testing all app features before rating. After the apps were rated, the raters met to discuss the app and the allocated score as a way to achieve agreement among raters and strengthen the scale. This discussion allowed for an identification of the similarities and differences in rating and, importantly, the strengths and weakness of each question in the scale, including clarity and specificity. During this process, the raters added and refined descriptors and examples for each item.

Phase 3: Reliability Analysis

The ABACUS version 2 was used by 3 raters (FM, SS, and MD) to review 50 physical activity apps. Physical activity apps were chosen for this phase not only because there are a large number of physical activity apps in the Apple iTunes store, providing a large choice for consumers but also because past reviewing [43] suggests that they represent a wide range in app quality. Apps were downloaded from the app store, and mirroring testing in phase 2, the authors spent approximately 10 min to 15 min reviewing all the features of the app.

Reliability of the scale was assessed using Krippendorff alpha. This allows for rating of ordinal data, can be used with an unlimited number of raters, and has been found to be superior to Cohen kappa [45,46]. Consistent with previous research, an alpha of more than .67 is used to indicate agreement [47], whereas, a negative alpha indicates less agreement than that would be expected by chance and suggests that there may have been inconsistencies in how measures were applied [48]. The internal consistency of the scale was calculated using Cronbach alpha. Interrater reliability was determined by interclass coefficient (ICC) [49]. Percentage agreement was also calculated.

Phase 4: Reliability Analysis 2

To investigate the discrepancies identified in phase 3, the same 3 raters (FM, SS, and MD) rated 5 unrated physical activity apps together. The apps were rated 1 at a time allowing for discussion of the results and for clarification of problem areas, specifically in item descriptions and examples. At the completion of this further moderation activity, an additional 20 apps were independently reviewed against ABACUS version 2, following the same procedure as phase 3.

Results

Phase 1

The search identified 593 unique papers. The abstracts and titles of all papers were reviewed, leaving 77 papers for full-text review. This review resulted in 50 papers that fully met the inclusion criteria and were included in this study (the list of resources is available in Table 1). To determine current best or common practice in app reviewing for behavior change, all scales used in the 50 papers identified were collected. For scales that were not provided as part of the manuscript or as a supplemental material, institution and academic sharing websites (such as Research.net) were searched. If the scale was not able to be located, the authors were emailed and a copy was requested. Only 2 scales [50,51] were unable to be obtained as the author had either moved on from that institution or there was no response to the email.

The scales identified in this systematic review were collated into a single document resulting in 1333 items (see Multimedia Appendix 1), with duplicates and questions present in the MARS removed, leaving 130 individual items (see Multimedia Appendix 1). Moreover, 2 authors (FM and SS) experienced in health promotion and health promoting apps reviewed the item pool. These authors had participated in the VicHealth Healthy Living Apps project [43], and each was experienced in rating hundreds of apps. The item pool was reviewed to identify or create items that were clear and based on previous work by these authors would be present in the highest quality apps [43].

From the 130 items, similar items were collapsed, for example, items that sought to identify avoidance or were collapsed with items that sought to minimize distraction; items that were presented as statements or single words were reworked into questions for ease of use. For example, 1 item that read "discrepancy between current behaviour and goal" was reworked to read "Does the app give the user the ability to quickly and easily understand the difference between current action and future goals?" This process resulted in an initial version of this scale, with 33 items that were categorized into 7 groups: (1) general, (2) goals, (3) feedback and monitoring, (4) knowledge and information, (5) actions, (6) rewards, and (7) environmental factors. These items formed the first version of the scale, the App Behavior Change Scale (ABACUS) version 1.

XSL•FO
RenderX

Table 1. Types and methods of evaluation.

Method	Location	Health condition	Reference
Data usage and user feedback	United Kingdom	Alcohol	Attwood et al [52]
Established evaluation checklist (Abraham and Michie 2008) [33]	New Zealand	Physical activity and dietary	Direito et al [21]
Established evaluation checklist (Abraham and & Michie 2008) [33]	The Netherlands	Health and Fitness	Middelweerd et al [20]
Established evaluation checklist (Abraham and Michie 2008) [33]	United States	Cancer	Vollmer et al [53]
Established evaluation checklist (CALO-RE)	United States	Physical activity	Conroy et al [10]
Established evaluation checklist (MARS[a] and Abroms, 2013 checklist)	Australia	Smoking	Thornton et al [28]
Established evaluation checklist (MARS)	New Zealand	Weight loss and smoking cessation	Patel et al [54]
Established evaluation checklist (MARS)	New Zealand	Travel and dietary behavior associated with health and environmental impact	Sullivan et al [44]
Established evaluation checklist (MARS) and self-developed evaluation checklist based on literature review	United States	Weight management	Bardus et al [55]
Established evaluation checklist (Michie et al) [32]	Ireland	Medication adherence	Morrissey et al [22]
Matched case-control trial	Australia	Physical activity	Kirwan et al [56]
Not discussed	Spain and United Kingdom	Iron-deficiency anemia, hearing loss, migraine, low vision, asthma, diabetes mellitus, osteoarthritis, and unipolar depressive disorders	Martínez-Pérez et al [2]
Self-developed checklist based on diabetes guidelines	United States	Diabetes	Nie et al [58]
Self-developed checklist based on epilepsy guidelines	Australia	Epilepsy	Pandher et al [59]
Self-developed checklist, established evaluation checklist (system usability scale)	United States	Chronic illness	Singh et al [60]
Self-developed evaluation checklist	Unites States	Smoking cessation	Abroms et al [61]
Self-developed evaluation checklist	Germany	Diabetes	Arnhold et al [1]
Self-developed evaluation checklist	United States	Weight management	Azar et al [4]
Self-developed evaluation checklist	Canada	Cancer	Bender et al [62]
Self-developed evaluation checklist	South Korea	Smoking cessation	Choi et al [63]
Self-developed evaluation checklist; user feedback	Norway	Diabetes	Chomutare et al [6]
Self-developed evaluation checklist; user feedback	United States	Alcohol	Cohn et al [50]
Self-developed evaluation checklist	United States	Diabetes and endocrinology	Eng et al [64]
Self-developed evaluation checklist	Unites States	Smoking cessation	Hoeppner et al [17]
Self-developed evaluation checklist	United Kingdom	Asthma	Huckvale et al [3]
Self-developed evaluation checklist	Canada	Headache	Hundert et al [65]
Self-developed evaluation checklist	United Kingdom	Melanoma	Kassianos et al [66]
Self-developed evaluation checklist; user feedback	United States	Hypertension	Kumar et al [67]
Self-developed evaluation checklist	Spain	Heart disease	Martínez-Perez et al [57]
Self-developed evaluation checklist; user feedback	United Kingdom	Breast cancer	Mobasheri et al [68]
Self-developed evaluation checklist	Australia	Bipolar disorder	Nicholas et al [69]
Self-developed evaluation checklist	Italy	Hearing	Paglialonga et al [70]
Self-developed evaluation checklist	United States	Weight-loss	Pagoto et al [71]

XSL•FO
RenderX

Method	Location	Health condition	Reference
Self-developed evaluation checklist; user feedback	United States	Cancer	Pandey et al [72]
Self-developed evaluation checklist; user feedback	Spain	Mindfulness	Plaza et al [73]
Self-developed evaluation checklist	United States	Mental health	Radovic et al [74]
Self-developed evaluation checklist; user feedback	United Kingdom	Pain	Reynoldson et al [8]
Self-developed evaluation checklist	Spain	HIV	Robustillo et al [75]
Self-developed evaluation checklist	United States	Health and wellness	Sama et al [76]
Self-developed evaluation checklist	Canada	Depression	Shen et al [77]
Self-developed evaluation checklist	United Kingdom	Smoking cessation	Ubhi et al [78]
Self-developed evaluation checklist; established evaluation checklist; user feedback	United States	Pediatric obesity	Wearing et al [79]
Self-developed evaluation checklist; user feedback	Australia	Alcohol	Weaver et al [80]
Self-developed evaluation checklist; user feedback	United States	Physical activity	Yang et al [81]
Self-developed evaluation checklist based on literature review	United States	Suicide prevention	Aguirre et al [82]
Self-developed evaluation checklist; established evaluation checklist (MARS)	United States	Pediatric medication adherence	Nguyen et al [83]
User feedback	Ireland	Physical activity	Casey et al [9]
User feedback	United Kingdom	Women's health	Derbyshire and Dancey [84]
User feedback	United States	Smoking	Ferron et al [85]
User feedback	Spain	Type 2 diabetes, obesity, and breast-feeding	García-Gómez et al [51]

[a]MARS: Medication Adherence Rating Scale.

Phase 2

This process resulted in the removal of 9 questions that were deemed to be unclear or were found to be duplicates or unnecessary. For example, in the initial scale, there were 4 separate items that outlined behavior costs, rewards, and encouragement. The authors' experience rating several hundreds of apps over a number of years, combined with the initial round of reviewing and discussion of this scale, allowed for the determination that more than one of these items were unlikely to be in the same app. As a result, these items were collapsed into 1 item: "Does the app provide general encouragement?" Other items were also removed at this point as it was determined that these questions were not relevant to behavior change, for example, a question about whether the app could be used without internet connection and 2 questions about expertise and consistency with national guidelines were collapsed into 1 question: "Was the app created with expertise and/or Does the app provides information that are consistent with national guidelines?"

The resulting scale contained 24 items. Following this, the scale was tested with 15 apps, 3 from each category: physical activity; healthy eating; alcohol; smoking; and mental well-being. Again, this process allowed for a refinement of the scale and resulted in several changes, including clarifying words and descriptors, reordering items, and combining other items, for example, 3 items relating to material, social, and self-reward or incentive were collapsed into a single item: "Does the app provide a material or social reward or incentive?" The authors' experience rating apps lead to the conclusion that it would unlikely that any 1 app would have more than 1 incentive or reward. Phase 2 resulted in the 22-item ABACUS version 2 with questions categorized into the following 4 categories: knowledge and information, goals and planning, feedback and monitoring, and actions.

At this stage, the ABACUS version 2 was sent to 7 external experts for their comment on content. These experts included 3 experts on mental well-being, 1 expert on alcohol and tobacco, 1 on physical activity, 1 on behavioral science, and 1 on health promotion. These experts were able to offer suggestions on language and terminology used, resulting in refinement of terminology and descriptors. For example, one of the reviewers suggested that the descriptor of item 1.4 (Does the app provide instruction on how to perform the behavior?) also includes video instructions (the app is clear in telling the person how to perform a behavior or preparatory behaviors, either verbally, through video, or in written form. Please note, the behavior that is seeking to be changed, not information on how to use the app). This version of the scale is presented in Table 2.

Phase 3

Phase 3 testing was conducted with 50 physical activity apps downloaded from the app store. All apps were rated independently by 3 reviewers against the ABACUS version 2, with ratings entered into Qualtrics to minimize user error. This phase found half of the questions to have high percentage agreement among reviewers (over 80%) with the scale overall reporting moderate interrater reliability (2-way mixed ICC=.69,

XSL•FO
RenderX

95% CI 0.52-0.82) and moderate internal consistency (Cronbach alpha=.71). However, some questions reported very low agreement. For example, question 4.3 “Does the app allow or encourage for practice or rehearsal, in addition to daily activities?” returned only an agreement of 51% with a negative Krippendorff alpha (alpha=−.01). Several other questions showed similarly low scores (see Table 2), and only 1 question achieved an alpha that would indicate agreement. These results prompted an additional round of discussion, and comparison was undertaken.

Phase 4

The initial discussion resulted in the collapsing of 2 *goal* questions into 1 from “Does the app allow for the setting of outcome (long-term) goals?” and “Does the app have the ability to set short and medium-term goals or a plan?” to “Does the app allow for the setting of goals?” Furthermore, a number of descriptors were reworded, and examples were provided for all questions. These changes resulted in ABACUS version 3 containing 21 questions (see Table 3 for final version of the scale).

This round of rating found over 80% of questions to have high percentage agreement among reviewers, with 3 questions scoring a Krippendorff alpha indicating agreement and a further 7 came close with alphas more than .5. The scale overall reported high interrater reliability (2-way mixed ICC=.91, 95% CI 0.81-0.97) and high internal consistency (Cronbach alpha=.93; see Table 4).

Table 2. Percentage agreement and reliability of App Behavior Change Scale version 2.

Item #	Measure	Phase 3 (50 apps)	
		Interrater reliability (Krippendorff alpha)	Percent agreement
1.1	Customize and personalize features	−.010	56
1.2	Consistent with national guidelines or created with expertise	.25	88
1.3	Baseline information	.45	73
1.4	Instruction on how to perform the behavior	.79	91
1.5	Information about the consequences of continuing and/or discontinuing behavior	.21	92
2.1	Willingness for behavior change	−.01	97
2.2	Goal setting	.22	83
2.3	Review goals, update, and change when necessary	.33	75
3.1	Understand the difference between current action and future goals	.60	84
3.2	Self-monitor behavior	.53	81
3.3	Share behaviors with others and/or allow for social comparison	.30	65
3.4	User feedback (in person or automatically)	.12	88
3.5	Export data	.16	77
3.6	Material or social reward or incentive	.19	66
3.7	General encouragement	.23	65
4.1	Reminders and/or prompts or cues for activity	.23	61
4.2	Encourage positive habit formation	.11	71
4.3	Practice or rehearsal, in addition to daily activities	−.01	51
4.4	Opportunity to plan for barriers	−.01	97
4.5	Restructuring the physical or social environment	−.01	97
4.6	Distraction or avoidance	−.02	95

XSL•FO
RenderX

Table 3. Final app behavior change scale, including examples. Yes = 1 No = 0

Scale: item number and question	Definition	Example or further information	Source of question (from Table 1)
**1. Knowledge and information**			
1.1 Does the app have the ability to customize and personalize some features? Yes	Elements of the app can be personalized through specific tools or functions that are specific to the individual using the app.	• To select a disease type from among several available and then to follow a specific path or set of tools or systems.<br>• To select to receive emails or texts of a specific nature.<br>• To choose "yes" or "no" to a specific capability of the app would be considered personalization.<br>• To create a personalized exercise plan.	[44,54]
1.2 Was the app created with expertise and/or Does the app provide information that is consistent with national guidelines? Yes	This would be found in the about section or generally in the app.	• Does the app suggest 30 min of exercise each day?<br>• Does it recommend 5 veg and 3 fruit?<br>• Does it seek to build resilience and promote help seeking?<br>• Is there any evidence that the app was created by an expert? (doctor/professional body/university)	[44,54]
1.3 Does the app ask for baseline information? Yes	This includes BMI[a], weight, smoking rate, exercise, or drinking behaviors	• This might be at the set-up phase or in a profile setting.	[28,85]
1.4 Does the app provide instruction on how to perform the behavior? Yes	The app is clear in telling the person how to perform a behavior or preparatory behaviors, either verbally, through video, or in written form.<br>NB: the behavior that is seeking to be changed, not information on how to use the app	• This could include showing person how to use gym equipment, sharing sample plans for action, instruction on suitable clothing, recipes, and general tips.	[20,21,22,81]
1.5 Does the app provide information about the consequences of continuing and/or discontinuing behavior? Yes	The app gives the user information about the consequences of behavior in general, this includes information about the relationship between the behavior and its possible or likely consequences in the general case. This information can be general or personalized.	• Consequences may include health, feelings, or cost consequences.	[22,81]
**2. Goals and planning**			
2.1 Does the app ask for willingness for behavior change? Yes	Is there a feature during setup where you describe how ready you are for behavior change?	• This may be in the form of a scale of readiness or in a question that asks the user to describe how ready you are.	[17,85]
2.2 Does the app allow for the setting of goals? Yes	The person is encouraged to make a behavioral resolution.<br>The person is encouraged to set a general goal that can be achieved by behavioral means. This includes subgoals or preparatory behaviors and/or specific contexts in which the behavior will be performed. The behavior in this technique will be directly related to or be a necessary condition for the target behavior.	• This is the explicit noting of a goal or choosing a goal from one provided within the app.	[20,21,40,44,54,55,81]

XSL•FO
RenderX

Scale: item number and question		Definition	Example or further information	Source of question (from Table 1)
2.3	Does the app have the ability to review goals, update, and change when necessary? Yes	Involves a review or analysis of the extent to which previously set behavioral goals (regardless of short or long) were achieved.	• This is where a goal can be changed. This allows people to act on previously set goals and then revise or adjust where needed.	[22,40,81]
**3. Feedback and monitoring**				
3.1	Does the app give the user the ability to quickly and easily understand the difference between current action and future goals? Yes	Allows user to see how they are tracking against a goal and to see the difference between what they want to do and what they are currently doing. This will give some feedback on where they are at and what they need to change to get to where they want to be.	• This could be in the form of a graph or some other visual describing how close the user is to meeting their goals.	[22,40,81]
3.2	Does the app have the ability to allow the user to easily self-monitor behavior? Yes	The app allows for a regular monitoring of the activity.	• Connects with watch that records daily steps that can be reviewed.<br>• Allows for easy logging of exercise or meditation?<br>• Allows for tracking of weight loss.<br>• Allows logging of daily alcoholic drinks or cigarettes.	[20,21]
3.3	Does the app have the ability to share behaviors with others (including social media or forums) and/or allow for social comparison? Yes	The app allows the person to share his or her behaviors on social media or in forums. This could also include a *buddy* system or a leaderboard.	• Share with Facebook or other socials<br>• Tell the user that they are doing x and at this time, other people like them are doing y	[4,20,21,22,85]
3.4	Does the app have the ability to give the user feedback—either from a person or automatically? Yes	The app is able to provide the person with feedback, comments, or data about their own recorded behavior. This might be automatic or could be personal.	• Does the app have a *coach* function?	[22,40,81]
3.5	Does the app have the ability to export data from app? No	The app allows for the export of information and progress to an external user.	• Export to a computer or to another user such as a doctor or fitness expert.<br>• Sharing to Facebook does not count.	[65]
3.6	Does the app provide a material or social reward or incentive? Yes	App provides rewards for attempts at achieving a behavioral goal. This might include efforts made toward achieving the behavior or progress made in preparatory steps toward the behavior or in achieving a goal.	• Financial, either in returning money that was not spent on, for example, cigarettes or in paying someone to engage in a specific activity.<br>• Social or public, for example, congratulating the person for each day that he or she meets his or her exercise target.	[22,40,81]
3.7	Does the app provide general encouragement? Yes	The app provides general encouragement and positive reinforcement on actions leading to the goal.	• This could include achievement badges or telling the user that they are a certain percentage closer to their goal.	[22,40,81]
**4. Actions**				

XSL•FO
RenderX

Scale: item number and question		Definition	Example or further information	Source of question (from Table 1)
4.1	Does the app have reminders and/or prompts or cues for activity? Yes	The app prompts the user to engage in the activity. The app has the ability to give notifications or reminders to cue the behavior.	• This could be like the apple watch reminding you to stand or a meditation app telling you to meditate now.	[20,21]
4.2	Does the app encourage positive habit formation? Yes	The app prompts explicit rehearsal and repetition of the behavior–not just tracking or logging.	• An example of this are the couch to 5 km apps that provide a training schedule.	[21,22,81]
4.3	Does the app allow or encourage for practice or rehearsal, in addition to daily activities? Yes	App does not have a lock on activities or a number that you cannot exceed daily.	• This would include allowing the user to undertake extra activities in a single day.	[20,21]
4.4	Does the app provide opportunity to plan for barriers? Yes	The app encourages the person to think about potential barriers and identify ways of overcoming them.	• Alcohol app might give strategies for a night out that would normally be a big night.	[55]
4.5	Does the app assist with or suggest restructuring the physical or social environment? Yes	The app prompts the person to alter the environment in ways so that it is more supportive of the target behavior.	• Might suggest locking up or throw away or their high-calorie snacks or take their running shoes to work.	[21,22,81]
4.6	Does the app assists with distraction or avoidance? Yes	The app gives suggestions and advice on how the person can avoid situations or distract themselves when trying to reach their goal.	• For example, a smoking cessation app may suggest that the user not drink coffee if this is typically combined with smoking behaviors that they are trying to cease.	[21,22,81]

Total Score = 4.75

XSL•FO
RenderX

Table 4. Percentage agreement and reliability of App Behavior Change Scale version 3.

Item #	Measure	Phase 4 (20 apps)	
		Interrater reliability (Krippendorff alpha)	Percent agreement
1.1	Customize and personalize features	.52	83
1.2	Consistent with national guidelines or created with expertise	.73	83
1.3	Baseline information	.79	90
1.4	Instruction on how to perform the behavior	.63	87
1.5	Information about the consequences of continuing and/or discontinuing behavior	−.02	93
2.1	Willingness for behavior change	0	97
2.2	Goal setting	.58	83
2.3	Review goals, update, and change when necessary	.38	80
3.1	Understand the difference between current action and future goals	.34	80
3.2	Self-monitor behavior	.62	83
3.3	Share behaviors with others and/or allow for social comparison	.73	87
3.4	User feedback (in person or automatically)	.26	67
3.5	Export data	.43	87
3.6	Material or social reward or incentive	.15	60
3.7	General encouragement	.54	77
4.1	Reminders and/or prompts or cues for activity	.61	80
4.2	Encourage positive habit formation	.28	63
4.3	Practice or rehearsal, in addition to daily activities	.05	80
4.4	Opportunity to plan for barriers	.31	93
4.5	Restructuring the physical or social environment	.57	93
4.6	Distraction or avoidance	1	100

Discussion

Principal Findings

This study reports on the creation of a scale (ABACUS) to measure the potential behavior change of smartphone apps. After conducting a systematic review to identify all research that has evaluated apps for behavior change, 133 items were identified and later modified after expert review to a final set of 21 items. The items within the scale are grouped into the following 4 categories: knowledge and information, goals and planning, feedback and monitoring, and actions. The ABACUS was reviewed by an expert panel and then tested first against 50 physical activity apps; however, because of concerns relating to moderate internal consistency and interrater reliability, an additional step of moderation was taken. This moderation saw the same raters come together to refine the scale, resulting in improved descriptors and the inclusion of examples for each question. Following this revision, the scale was used to rate an additional 20 apps. This round of ratings resulted in a high internal consistency and interrater reliability. Although previous studies evaluating smartphone apps have focused largely on features available in apps [21] or behavior change techniques through a self-developed evaluation checklist [4,10], the ABACUS provides researchers with a reliable and valid instrument to evaluate apps based on their behavior change potential.

This scale will allow researchers to investigate the behavior change potential of a large number of apps reasonably quickly. This is important, as the fast-moving pace of app technology means that although randomized controlled trials (RCTs) remain important in understanding the impacts of individual apps on behavior [86], it has been suggested that the RCT may not be the most appropriate method to generate evidence around mobile apps [28]. RCTs can take a significant amount of time in planning and design meaning that by the time the RCT is available for publication, the information is no longer current [28]. The scale developed in this research is not a replacement for an RCT but rather will allow researchers and consumers to understand the behavior change potential of an app in the absence of an RCT.

The MARS [42], a 23-item tool included 5 subscales for measuring app quality: engagement, functionality, aesthetics, information, and app subjective quality, with questions such as target age group, ease of navigation, or aesthetics can be used in conjunction with the ABACUS. The MARS is a useful tool in understanding the aesthetic and functional appeal of an app. When used together, the MARS and the ABACUS will allow researchers to provide users with 2 scores for each app: 1 that

XSL•FO
RenderX

measures app quality and 1 that measures potential for behavior change.

This study is only a starting point in the identification and interpretation of the behavior change potential of smartphone apps. This study only reports on the validation and reliability of physical activity apps, and as such, further testing of the scale should be conducted on additional health areas such as smoking, alcohol, and nutrition, as it is possible that different items may be important for these health areas. Furthermore, a more detailed investigation into the relative scores of apps will need to be undertaken. This will allow for an understanding of the importance of the overall score assigned to each app. At present, this scale is best understood as providing a continuous score rather than specific cut-off points. However, this is not to say that with more investigation and testing that clear scores could not provide a consumer with a numerical rating reflecting a behavioral outcome. This study has not purported to demonstrate correlation between an app's score and the health outcome; however, this scale could be used in future along with a more detailed study of individual apps and the behavior change outcomes in using them.

ABACUS has good interrater reliability and is a valid tool for evaluating the potential behavior change in smartphone apps. The validation and reliability testing of ABACUS contributes to the literature by providing a standardized method of evaluating smartphone apps for behavior change.

Limitations

Although this scale shows good reliability and validity, there are several limitations that need to be addressed. The first is that we have not sought to investigate criterion validity. The scale presented in this paper seeks to measure the theoretical behavior change potential of apps; and therefore, we do not seek to investigate the relationship between actual features of apps and behavioral outcomes. This scale has not been designed for this type of activity, so we leave this up to others to identify an appropriate method for such an investigation. Although reducing the numbers of items on the scale facilitates faster rating, there is a risk that removal of duplicate items and streamlining these items into 1 binary response may inflate a score. For example, by collapsing all *goal*-setting activities into 1 item, this scale recognizes apps that have any goals-setting ability, rather than the strength of that ability—a feature found in the behavior change taxonomy. Furthermore, there is a risk that by collapsing items that record starting a positive behavior with stopping a negative behavior, we may be missing a key aspect of behavior change. These decisions were made based on the authors' experience of rating apps with an understanding that a single app will not include both of these features, and as such, in seeking to provide a succinct scale, it makes more sense to only measure 1 outcome. Like other similar studies [42], this study highlights the importance of rater's knowledge of apps when completing such evaluations and with moderating 5 to 10 apps at the beginning of the process as a team is important to ensure a robust score. In addition, similar to other studies, raters in this study spent 10 min to 15 min with the app to become familiar before completing the evaluation. This time spent using the app is consistent with other studies that seek to review apps, as a longer time under review is not realistic [42,87]. Finally, 1 key limitation of this study is that the scale has been validated on physical activity apps. Although this scale seeks to be used in the future for other health behaviors, at this point in time, we are only confident that it can be used to rate the health behavior potential of physical activity apps. Other health behaviors will need to be investigated in future studies.

Conclusions

The ABACUS is a reliable tool that can be used to determine the behavior change potential of apps. This instrument fills a gap by allowing the evaluation of a large number of apps to be standardized across a range of health categories. This scale can be used by teams to rate apps that seek to promote behavior change, allowing for high-quality apps to then be recommended to the general public.

Acknowledgments

This study was partly funded by the Victorian Health Promotion Foundation.

Conflicts of Interest

None declared.

Multimedia Appendix 1

Items from systematic review.

[XLSX File (Microsoft Excel File), 80KB - mhealth_v7i1e11130_app1.xlsx]

References

1. Arnhold M, Quade M, Kirch W. Mobile applications for diabetics: a systematic review and expert-based usability evaluation considering the special requirements of diabetes patients age 50 years or older. J Med Internet Res 2014 Apr 09;16(4):e104 [FREE Full text] [doi: 10.2196/jmir.2968] [Medline: 24718852]
2. Martínez-Pérez B, de la Torre-Díez I, López-Coronado M. Mobile health applications for the most prevalent conditions by the World Health Organization: review and analysis. J Med Internet Res 2013 Jun 14;15(6):e120 [FREE Full text] [doi: 10.2196/jmir.2600] [Medline: 23770578]

XSL•FO
RenderX

3. Huckvale K, Car M, Morrison C, Car J. Apps for asthma self-management: a systematic assessment of content and tools. BMC Med 2012 Nov 22;10:144 [FREE Full text] [doi: 10.1186/1741-7015-10-144] [Medline: 23171675]
4. Azar KM, Lesser LI, Laing BY, Stephens J, Aurora MS, Burke LE, et al. Mobile applications for weight management: theory-based content analysis. Am J Prev Med 2013 Nov;45(5):583-589. [doi: 10.1016/j.amepre.2013.07.005] [Medline: 24139771]
5. Research2Guidance. 325,000 mobile health apps available in 2017 – Android now the leading mHealth platform URL: https://research2guidance.com/325000-mobile-health-apps-available-in-2017/ [accessed 2018-02-07] [WebCite Cache ID 6x2aCVSet]
6. Chomutare T, Fernandez-Luque L, Arsand E, Hartvigsen G. Features of mobile diabetes applications: review of the literature and analysis of current applications compared against evidence-based guidelines. J Med Internet Res 2011 Sep 22;13(3):e65 [FREE Full text] [doi: 10.2196/jmir.1874] [Medline: 21979293]
7. Cardoş RA, Şoflău R, Gherman A, Sucală M, Chiorean A. A mobile intervention for core needle biopsy related pain and anxiety: a usability study. J Evid-Based Psychot 2017 Mar 01;17(1):21-30. [doi: 10.24193/jebp.2017.1.2]
8. Reynoldson C, Stones C, Allsop M, Gardner P, Bennett MI, Closs SJ, et al. Assessing the quality and usability of smartphone apps for pain self-management. Pain Med 2014 Jun;15(6):898-909. [doi: 10.1111/pme.12327] [Medline: 24422990]
9. Casey M, Hayes PS, Glynn F, OLaighin G, Heaney D, Murphy AW, et al. Patients' experiences of using a smartphone application to increase physical activity: the SMART MOVE qualitative study in primary care. Br J Gen Pract 2014 Aug;64(625):e500-e508 [FREE Full text] [doi: 10.3399/bjgp14X680989] [Medline: 25071063]
10. Conroy DE, Yang C, Maher JP. Behavior change techniques in top-ranked mobile apps for physical activity. Am J Prev Med 2014 Jun;46(6):649-652. [doi: 10.1016/j.amepre.2014.01.010] [Medline: 24842742]
11. Lieffers J, Hanning RM. Dietary assessment and self-monitoring with nutrition applications for mobile devices. Can J Diet Pract Res 2012;73(3):e253-e260. [doi: 10.3148/73.3.2012.e253] [Medline: 22958633]
12. Tonkin E, Brimblecombe J, Wycherley T. Characteristics of smartphone applications for nutrition improvement in community settings: a scoping review. Adv Nutr 2017 Mar;8(2):308-322 [FREE Full text] [doi: 10.3945/an.116.013748] [Medline: 28298274]
13. Grist R, Porter J, Stallard P. Mental health mobile apps for preadolescents and adolescents: a systematic review. J Med Internet Res 2017 Dec 25;19(5):e176 [FREE Full text] [doi: 10.2196/jmir.7332] [Medline: 28546138]
14. Lui J, Marcus D, Barry C. Evidence-based apps? A review of mental health mobile applications in a psychotherapy context. Prof Psychol Res Pr 2017;48(3):199-210. [doi: 10.1037/pro0000122]
15. Lim M, Hocking J, Aitken C, Fairley C, Jordan L, Lewis J, et al. Impact of text and email messaging on the sexual health of young people: a randomised controlled trial. J Epidemiol Community Health 2012 Jan;66(1):69-74. [doi: 10.1136/jech.2009.100396] [Medline: 21415232]
16. Hall A, Cole-Lewis H, Bernhardt J. Mobile text messaging for health: a systematic review of reviews. Annu Rev Public Health 2015 Mar 18;36:393-415 [FREE Full text] [doi: 10.1146/annurev-publhealth-031914-122855] [Medline: 25785892]
17. Hoeppner BB, Hoeppner SS, Seaboyer L, Schick MR, Wu GW, Bergman BG, et al. How smart are smartphone apps for smoking cessation? A content analysis. Nicotine Tob Res 2016 May;18(5):1025-1031 [FREE Full text] [doi: 10.1093/ntr/ntv117] [Medline: 26045249]
18. Hoeppner BB, Schick MR, Kelly LM, Hoeppner SS, Bergman B, Kelly JF. There is an app for that - Or is there? A content analysis of publicly available smartphone apps for managing alcohol use. J Subst Abuse Treat 2017 Nov;82:67-73. [doi: 10.1016/j.jsat.2017.09.006] [Medline: 29021117]
19. Crane D, Garnett C, Brown J, West R, Michie S. Behavior change techniques in popular alcohol reduction apps: content analysis. J Med Internet Res 2015 May 14;17(5):e118 [FREE Full text] [doi: 10.2196/jmir.4060] [Medline: 25977135]
20. Middelweerd A, Mollee J, van der Wal CN, Brug J, Te Velde SJ. Apps to promote physical activity among adults: a review and content analysis. Int J Behav Nutr Phys Act 2014 Jul 25;11:97 [FREE Full text] [doi: 10.1186/s12966-014-0097-9] [Medline: 25059981]
21. Direito A, Dale L, Shields E, Dobson R, Whittaker R, Maddison R. Do physical activity and dietary smartphone applications incorporate evidence-based behaviour change techniques? BMC Public Health 2014 Jun 25;14:646 [FREE Full text] [doi: 10.1186/1471-2458-14-646] [Medline: 24965805]
22. Morrissey E, Corbett T, Walsh J, Molloy G. Behavior change techniques in apps for medication adherence: a content analysis. Am J Prev Med 2016 May;50(5):e143-e146. [doi: 10.1016/j.amepre.2015.09.034] [Medline: 26597504]
23. Roberts A, Fisher A, Smith L, Heinrich M, Potts H. Digital health behaviour change interventions targeting physical activity and diet in cancer survivors: a systematic review and meta-analysis. J Cancer Surviv 2017 Dec;11(6):704-719 [FREE Full text] [doi: 10.1007/s11764-017-0632-1] [Medline: 28779220]
24. Bakker D, Kazantzis N, Rickwood D, Rickard N. A randomized controlled trial of three smartphone apps for enhancing public mental health. Behav Res Ther 2018 Oct;109:75-83. [doi: 10.1016/j.brat.2018.08.003] [Medline: 30125790]
25. Flores Mateo G, Granado-Font E, Ferré-Grau C, Montaña-Carreras X. Mobile phone apps to promote weight loss and increase physical activity: a systematic review and meta-analysis. J Med Internet Res 2015 Nov 10;17(11):e253 [FREE Full text] [doi: 10.2196/jmir.4836] [Medline: 26554314]

XSL•FO
RenderX

26. Flaherty S, McCarthy M, Collins A, McAuliffe F. Can existing mobile apps support healthier food purchasing behaviour? Content analysis of nutrition content, behaviour change theory and user quality integration. Public Health Nutr 2018 Dec;21(2):288-298. [doi: 10.1017/S1368980017002889] [Medline: 29081322]
27. Ptakauskaite N, Cox AL, Berthouze N. Knowing what you're doing or knowing what to do: how stress management apps support reflection and behaviour change. In: CHI EA '18 Extended Abstracts of the 2018 CHI Conference on Human Factors in Computing Systems. 2018 Presented at: CHI EA '18; April 21 - 26, 2018; Montreal QC, Canada. [doi: 10.1145/3170427.3188648]
28. Thornton L, Quinn C, Birrell L, Guillaumier A, Shaw B, Forbes E, et al. Free smoking cessation mobile apps available in Australia: a quality review and content analysis. Aust N Z J Public Health 2017 Dec;41(6):625-630. [doi: 10.1111/1753-6405.12688] [Medline: 28749591]
29. Reyes A, Qin P, Brown CA. A standardized review of smartphone applications to promote balance for older adults. Disabil Rehabil 2018 Dec;40(6):690-696. [doi: 10.1080/09638288.2016.1250124] [Medline: 27868438]
30. Weekly T, Walker N, Beck J, Akers S, Weaver M. A review of apps for calming, relaxation, and mindfulness interventions for pediatric palliative care patients. Children (Basel) 2018 Jan 26;5(2) [FREE Full text] [doi: 10.3390/children5020016] [Medline: 29373515]
31. McKay F, Cheng C, Wright A, Shill J, Stephens H, Uccellini M. Evaluating mobile phone applications for health behaviour change: a systematic review. J Telemed Telecare 2018 Jan;24(1):22-30. [doi: 10.1177/1357633X16673538] [Medline: 27760883]
32. Michie S, Richardson M, Johnston M, Abraham C, Francis J, Hardeman W, et al. The behavior change technique taxonomy (v1) of 93 hierarchically clustered techniques: building an international consensus for the reporting of behavior change interventions. Ann Behav Med 2013 Aug;46(1):81-95. [doi: 10.1007/s12160-013-9486-6] [Medline: 23512568]
33. Abraham C, Michie S. A taxonomy of behavior change techniques used in interventions. Health Psychol 2008 May;27(3):379-387. [doi: 10.1037/0278-6133.27.3.379] [Medline: 18624603]
34. Michie S, Abraham C, Whittington C, McAteer J, Gupta S. Effective techniques in healthy eating and physical activity interventions: a meta-regression. Health Psychol 2009 Nov;28(6):690-701. [doi: 10.1037/a0016136] [Medline: 19916637]
35. Dombrowski S, Sniehotta F, Avenell A, Johnston M, MacLennan G, Araújo-Soares V. Identifying active ingredients in complex behavioural interventions for obese adults with obesity-related co-morbidities or additional risk factors for co-morbidities: a systematic review. Health Psychol Rev 2012 Mar;6(1):7-32. [doi: 10.1080/17437199.2010.513298]
36. Burke L, Wang J, Sevick M. Self-monitoring in weight loss: a systematic review of the literature. J Am Diet Assoc 2011 Jan;111(1):92-102 [FREE Full text] [doi: 10.1016/j.jada.2010.10.008] [Medline: 21185970]
37. Michie S, Whittington C, Hamoudi Z, Zarnani F, Tober G, West R. Identification of behaviour change techniques to reduce excessive alcohol consumption. Addiction 2012 Aug;107(8):1431-1440. [doi: 10.1111/j.1360-0443.2012.03845.x] [Medline: 22340523]
38. Cook S, Palmer M, Shuler FD. Smartphone mobile applications to enhance diagnosis of skin cancer: a guide for the rural practitioner. W V Med J 2015;111(5):22-28. [Medline: 26521532]
39. Boyle L, Grainger R, Hall RM, Krebs JD. Use of and beliefs about mobile phone apps for diabetes self-management: surveys of people in a hospital diabetes clinic and diabetes health professionals in New Zealand. JMIR Mhealth Uhealth 2017 Jun 30;5(6):e85 [FREE Full text] [doi: 10.2196/mhealth.7263] [Medline: 28666975]
40. Michie S, Ashford S, Sniehotta FF, Dombrowski SU, Bishop A, French DP. A refined taxonomy of behaviour change techniques to help people change their physical activity and healthy eating behaviours: the CALO-RE taxonomy. Psychol Health 2011 Nov;26(11):1479-1498. [doi: 10.1080/08870446.2010.540664] [Medline: 21678185]
41. Michie S, Johnston M, Francis J, Hardeman W, Eccles M. From theory to intervention: mapping theoretically derived behavioural determinants to behaviour change techniques. Appl Psychol 2008;57(4):660-680. [doi: 10.1111/j.1464-0597.2008.00341.x]
42. Stoyanov S, Hides L, Kavanagh D, Zelenko O, Tjondronegoro D, Mani M. Mobile app rating scale: a new tool for assessing the quality of health mobile apps. JMIR Mhealth Uhealth 2015 Mar 11;3(1):e27 [FREE Full text] [doi: 10.2196/mhealth.3422] [Medline: 25760773]
43. VicHealth. VicHealth Healthy Living Apps project 2016 URL: https://www.vichealth.vic.gov.au/media-and-resources/vichealth-apps/healthy-living-apps [accessed 2018-02-08] [WebCite Cache ID 6x4GR3CxS]
44. Sullivan R, Marsh S, Halvarsson J, Holdsworth M, Waterlander W, Poelman M, et al. Smartphone apps for measuring human health and climate change co-benefits: a comparison and quality rating of available apps. JMIR Mhealth Uhealth 2016 Dec 19;4(4):e135 [FREE Full text] [doi: 10.2196/mhealth.5931] [Medline: 27993762]
45. Bock MA, Krippendorff K. The Content Analysis Reader. London: SAGE Publications; 2008.
46. Hayes A, Krippendorff K. Answering the call for a standard reliability measure for coding data. Commun Methods Meas 2007 Apr;1(1):77-89. [doi: 10.1080/19312450709336664]
47. Krippendorff K. Content Analysis: An Introduction to Its Methodology. London: Sage Publications; 1980.
48. Krippendorff K. Reliability in content analysis. Human Comm Res 2004 Jul;30(3):411-433. [doi: 10.1111/j.1468-2958.2004.tb00738.x]

XSL•FO
RenderX

49. Shrout PE, Fleiss JL. Intraclass correlations: uses in assessing rater reliability. Psychol Bull 1979 Mar;86(2):420-428. [Medline: 18839484]
50. Cohn AM, Hunter-Reel D, Hagman BT, Mitchell J. Promoting behavior change from alcohol use through mobile technology: the future of ecological momentary assessment. Alcohol Clin Exp Res 2011 Dec;35(12):2209-2215 [FREE Full text] [doi: 10.1111/j.1530-0277.2011.01571.x] [Medline: 21689119]
51. García-Gómez JM, de la Torre-Díez I, Vicente J, Robles M, López-Coronado M, Rodrigues J. Analysis of mobile health applications for a broad spectrum of consumers: a user experience approach. Health Informatics J 2014 Mar;20(1):74-84. [doi: 10.1177/1460458213479598] [Medline: 24550566]
52. Attwood S, Parke H, Larsen J, Morton K. Using a mobile health application to reduce alcohol consumption: a mixed-methods evaluation of the drinkaware track & calculate units application. BMC Public Health 2017 Dec 17;17(1):394 [FREE Full text] [doi: 10.1186/s12889-017-4358-9] [Medline: 28511698]
53. Vollmer DD, Fair K, Hong YA, Beaudoin CE, Pulczinski J, Ory MG. Apps seeking theories: results of a study on the use of health behavior change theories in cancer survivorship mobile apps. JMIR Mhealth Uhealth 2015;3(1):e31 [FREE Full text] [doi: 10.2196/mhealth.3861] [Medline: 25830810]
54. Patel R, Sulzberger L, Li G, Mair J, Morley H, Shing MN, et al. Smartphone apps for weight loss and smoking cessation: quality ranking of 120 apps. N Z Med J 2015 Sep 04;128(1421):73-76. [Medline: 26370762]
55. Bardus M, van Beurden SB, Smith J, Abraham C. A review and content analysis of engagement, functionality, aesthetics, information quality, and change techniques in the most popular commercial apps for weight management. Int J Behav Nutr Phys Act 2016 Mar 10;13:35 [FREE Full text] [doi: 10.1186/s12966-016-0359-9] [Medline: 26964880]
56. Kirwan M, Duncan M, Vandelanotte C, Mummery W. Using smartphone technology to monitor physical activity in the 10,000 Steps program: a matched case-control trial. J Med Internet Res 2012 Apr 20;14(2):e55 [FREE Full text] [doi: 10.2196/jmir.1950] [Medline: 22522112]
57. Martínez-Pérez B, de la Torre-Díez I, López-Coronado M, Herreros-González J. Mobile apps in cardiology: review. JMIR Mhealth Uhealth 2013 Jul 24;1(2):e15 [FREE Full text] [doi: 10.2196/mhealth.2737] [Medline: 25098320]
58. Nie L, Xie B, Yang Y, Shan YM. Characteristics of Chinese m-Health applications for diabetes self-management. Telemed J E Health 2016 Dec;22(7):614-619 [FREE Full text] [doi: 10.1089/tmj.2015.0184] [Medline: 27171016]
59. Pandher PS, Bhullar KK. Smartphone applications for seizure management. Health Informatics J 2016 Dec;22(2):209-220. [doi: 10.1177/1460458214540906] [Medline: 25038202]
60. Singh K, Drouin K, Newmark LP, Lee J, Faxvaag A, Rozenblum R, et al. Many mobile health apps target high-need, high-cost populations, but gaps remain. Health Aff (Millwood) 2016 Dec 01;35(12):2310-2318. [doi: 10.1377/hlthaff.2016.0578] [Medline: 27920321]
61. Abroms LC, Lee Westmaas J, Bontemps-Jones J, Ramani R, Mellerson J. A content analysis of popular smartphone apps for smoking cessation. Am J Prev Med 2013 Dec;45(6):732-736 [FREE Full text] [doi: 10.1016/j.amepre.2013.07.008] [Medline: 24237915]
62. Bender J, Yue R, To M, Deacken L, Jadad A. A lot of action, but not in the right direction: systematic review and content analysis of smartphone applications for the prevention, detection, and management of cancer. J Med Internet Res 2013 Dec 23;15(12):e287 [FREE Full text] [doi: 10.2196/jmir.2661] [Medline: 24366061]
63. Choi J, Noh GY, Park DJ. Smoking cessation apps for smartphones: content analysis with the self-determination theory. J Med Internet Res 2014 Feb 12;16(2):e44 [FREE Full text] [doi: 10.2196/jmir.3061] [Medline: 24521881]
64. Eng DS, Lee JM. The promise and peril of mobile health applications for diabetes and endocrinology. Pediatr Diabetes 2013 Jun;14(4):231-238 [FREE Full text] [doi: 10.1111/pedi.12034] [Medline: 23627878]
65. Hundert A, Huguet A, McGrath P, Stinson J, Wheaton M. Commercially available mobile phone headache diary apps: a systematic review. JMIR Mhealth Uhealth 2014 Aug 19;2(3):e36 [FREE Full text] [doi: 10.2196/mhealth.3452] [Medline: 25138438]
66. Kassianos A, Emery J, Murchie P, Walter F. Smartphone applications for melanoma detection by community, patient and generalist clinician users: a review. Br J Dermatol 2015 Jun;172(6):1507-1518. [doi: 10.1111/bjd.13665] [Medline: 25600815]
67. Kumar N, Khunger M, Gupta A, Garg N. A content analysis of smartphone-based applications for hypertension management. J Am Soc Hypertens 2015 Feb;9(2):130-136. [doi: 10.1016/j.jash.2014.12.001] [Medline: 25660364]
68. Mobasheri MH, Johnston M, King D, Leff D, Thiruchelvam P, Darzi A. Smartphone breast applications - what's the evidence? Breast 2014 Oct;23(5):683-689. [doi: 10.1016/j.breast.2014.07.006] [Medline: 25153432]
69. Nicholas J, Larsen M, Proudfoot J, Christensen H. Mobile apps for bipolar disorder: a systematic review of features and content quality. J Med Internet Res 2015 Aug 17;17(8):e198 [FREE Full text] [doi: 10.2196/jmir.4581] [Medline: 26283290]
70. Paglialonga A, Pinciroli F, Tognola G. The ALFA4Hearing model (At-a-Glance Labeling for Features of Apps for Hearing Health Care) to characterize mobile apps for hearing health care. Am J Audiol 2017 Oct 12;26(3S):408-425. [doi: 10.1044/2017_AJA-16-0132] [Medline: 29049624]
71. Pagoto S, Schneider K, Jojic M, DeBiasse M, Mann D. Evidence-based strategies in weight-loss mobile apps. Am J Prev Med 2013 Nov;45(5):576-582. [doi: 10.1016/j.amepre.2013.04.025] [Medline: 24139770]

XSL•FO
RenderX

72. Pandey A, Hasan S, Dubey D, Sarangi S. Smartphone apps as a source of cancer information: changing trends in health information-seeking behavior. J Cancer Educ 2013 Mar;28(1):138-142. [doi: 10.1007/s13187-012-0446-9] [Medline: 23275239]
73. Plaza I, Demarzo MM, Herrera-Mercadal P, García-Campayo J. Mindfulness-based mobile applications: literature review and analysis of current features. JMIR Mhealth Uhealth 2013 Nov 01;1(2):e24 [FREE Full text] [doi: 10.2196/mhealth.2733] [Medline: 25099314]
74. Radovic A, Vona PL, Santostefano AM, Ciaravino S, Miller E, Stein BD. Smartphone applications for mental health. Cyberpsychol Behav Soc Netw 2016 Jul;19(7):465-470 [FREE Full text] [doi: 10.1089/cyber.2015.0619] [Medline: 27428034]
75. Robustillo Cortés Mde L, Cantudo Cuenca MR, Morillo Verdugo R, Calvo Cidoncha E. High quantity but limited quality in healthcare applications intended for HIV-infected patients. Telemed J E Health 2014 Aug;20(8):729-735. [doi: 10.1089/tmj.2013.0262] [Medline: 24849001]
76. Sama PR, Eapen ZJ, Weinfurt KP, Shah BR, Schulman KA. An evaluation of mobile health application tools. JMIR Mhealth Uhealth 2014 May 01;2(2):e19 [FREE Full text] [doi: 10.2196/mhealth.3088] [Medline: 25099179]
77. Shen N, Levitan M, Johnson A, Bender J, Hamilton-Page M, Jadad A, et al. Finding a depression app: a review and content analysis of the depression app marketplace. JMIR Mhealth Uhealth 2015 Feb 16;3(1):e16 [FREE Full text] [doi: 10.2196/mhealth.3713] [Medline: 25689790]
78. Ubhi HK, Kotz D, Michie S, van Schayck OC, Sheard D, Selladurai A, et al. Comparative analysis of smoking cessation smartphone applications available in 2012 versus 2014. Addict Behav 2016 Jul;58:175-181 [FREE Full text] [doi: 10.1016/j.addbeh.2016.02.026] [Medline: 26950256]
79. Wearing JR, Nollen N, Befort C, Davis AM, Agemy CK. iPhone app adherence to expert-recommended guidelines for pediatric obesity prevention. Child Obes 2014 Apr;10(2):132-144 [FREE Full text] [doi: 10.1089/chi.2013.0084] [Medline: 24655230]
80. Weaver ER, Horyniak DR, Jenkinson R, Dietze P, Lim MS. “Let's get wasted!” and other apps: characteristics, acceptability, and use of alcohol-related smartphone applications. JMIR Mhealth Uhealth 2013 Jun 25;1(1):e9 [FREE Full text] [doi: 10.2196/mhealth.2709] [Medline: 25100681]
81. Yang C, Maher JP, Conroy DE. Implementation of behavior change techniques in mobile applications for physical activity. Am J Prev Med 2015 Apr;48(4):452-455. [doi: 10.1016/j.amepre.2014.10.010] [Medline: 25576494]
82. Aguirre R, McCoy M, Roan M. Development guidelines from a study of suicide prevention mobile spplications (Apps). J Technol Hum Serv 2013 Jul;31(3):269-293. [doi: 10.1080/15228835.2013.814750]
83. Nguyen E, Bugno L, Kandah C, Plevinsky J, Poulopoulos N, Wojtowicz A, et al. Is there a good app for that? Evaluating m-Health apps for strategies that promote pediatric medication adherence. Telemed J E Health 2016 Dec;22(11):929-937. [doi: 10.1089/tmj.2015.0211] [Medline: 27070837]
84. Derbyshire E, Dancey D. Smartphone Medical Applications for Women's Health: What Is the Evidence-Base and Feedback? Int J Telemed Appl 2013;2013 [FREE Full text] [doi: 10.1155/2013/782074] [Medline: 24454354]
85. Ferron J, Brunette M, Geiger P, Marsch L, Adachi-Mejia A, Bartels S. Mobile phone apps for smoking cessation: quality and usability among smokers with psychosis. JMIR Hum Factors 2017 Mar 03;4(1):e7 [FREE Full text] [doi: 10.2196/humanfactors.5933] [Medline: 28258047]
86. Vaz CL, Suthar AG, Pousti BT, Aye SM, Williams KJ, Zhao H. A smartphone app–based lifestyle intervention promotes weight loss—results of a prospective, randomized, controlled clinical trial (RCT). Diabetes 2018;67(Supplement 1). [doi: 10.2337/db18-2070-P]
87. Powell A, Torous J, Chan S, Raynor G, Shwarts E, Shanahan M, et al. Interrater reliability of mHealth app rating measures: analysis of top depression and smoking cessation apps. JMIR Mhealth Uhealth 2016 Feb 10;4(1):e15 [FREE Full text] [doi: 10.2196/mhealth.5176] [Medline: 26863986]

Abbreviations

ABACUS: App Behavior Change Scale
ICC: interclass coefficient
MARS: Mobile App Rating Scale
RCT: randomized controlled trial
VicHealth: Victorian Health Promotion Foundation

XSL•FO
RenderX

Edited by G Eysenbach; submitted 24.05.18; peer-reviewed by K Stawarz, A Crandall, A Nguyen, H Potts; comments to author 04.08.18; revised version received 29.10.18; accepted 29.10.18; published 25.01.19

Please cite as:
McKay FH, Slykerman S, Dunn M
The App Behavior Change Scale: Creation of a Scale to Assess the Potential of Apps to Promote Behavior Change
JMIR Mhealth Uhealth 2019;7(1):e11130
URL: http://mhealth.jmir.org/2019/1/e11130/
doi:10.2196/11130
PMID:

©Fiona H McKay, Sarah Slykerman, Matthew Dunn. Originally published in JMIR Mhealth and Uhealth (http://mhealth.jmir.org), 25.01.2019. This is an open-access article distributed under the terms of the Creative Commons Attribution License (https://creativecommons.org/licenses/by/4.0/), which permits unrestricted use, distribution, and reproduction in any medium, provided the original work, first published in JMIR mhealth and uhealth, is properly cited. The complete bibliographic information, a link to the original publication on http://mhealth.jmir.org/, as well as this copyright and license information must be included.

XSL•FO
RenderX
